# Characterization and Neuroprotection Potential of Seleno-Polymannuronate

**DOI:** 10.3389/fphar.2020.00021

**Published:** 2020-02-20

**Authors:** Decheng Bi, Xiaofan Li, Tong Li, Xiuting Li, Zhijian Lin, Lijun Yao, Hui Li, Hong Xu, Zhangli Hu, Zhenqing Zhang, Qiong Liu, Xu Xu

**Affiliations:** ^1^Shenzhen Key Laboratory of Marine Bioresource and Eco-environmental Science, Guangdong Provincial Key Laboratory for Plant Epigenetics, College of Life Sciences and Oceanography, Shenzhen University, Shenzhen, China; ^2^Beijing Advanced Innovation Center for Food Nutrition and Human Health, Beijing Technology and Business University (BTBU), Beijing, China; ^3^Department of Neurology, Peking University Shenzhen Hospital, Shenzhen, China; ^4^College of Pharmaceutical Sciences, Soochow University, Suzhou, China

**Keywords:** alginate, seleno-polymannuronate, anti-apoptosis, neuroprotection, N2a-sw cells

## Abstract

Seleno-polymannuronate (Se-PM) was prepared from alginate-derived polymannuronate (PM) through a sulfation followed by a selenylation replacement reaction. The organic selenium content of Se-PM was 437.25 μg/g and its average molecular weight was 2.36 kDa. The neuroprotection effect of Se-PM and corresponding molecular mechanisms were investigated. Our results showed that, comparing to both sulfated PM (S-PM) and PM, Se-PM remarkably inhibited the aggregation of Aβ_1–42_ oligomer *in vitro* and significantly reduced the APP and BACE1 protein expression in N2a-sw cells, highlighting the critical function of the selenium presented in Se-PM. Moreover, Se-PM decreased the expression of cytochrome c and the ratio of Bax to Bcl-2, and enhanced the mitochondrial membrane potential in N2a-sw cells. These results suggested that Se-PM treatment can markedly inhibit N2a-sw cell apoptosis and promote N2a-sw cell survival and that Se-PM might be a potential therapeutic agent for the prevention of neurodegeneration owing to its remarkable neuroprotection effect.

## Introduction

Alginate, a naturally acidic polysaccharide, consists of alternating β-D-mannuronic acid and α-L-guluronic acid moieties connected with 1,4-glycosidic linkages, and it is common in various edible brown seaweeds (Haug et al., 1967). Alginate is widely used as a biopolymer in the food, cosmetic, pharmaceutical, and textile industries due to its stability, biodegradability, and low toxicity (Lee and Mooney, 2012). Alginate and its derivatives have been shown to have numerous biological and pharmacological activities, including neuroprotective (Zhou et al., 2015a), anti-inflammatory (Zhou et al., 2015b), immunostimulatory (Xu et al., 2014a; Xu et al., 2014b; Bi et al., 2017; Fang et al., 2017), and anti-oxidant (Tusi et al., 2011) effects. Polymannuronate (PM) is an acidic polysaccharide prepared from alginate *via* hydrolyzation by hydrogen chloride (HCl) and separation by fractionation at pH 2.85 (Haug et al., 1967). PM has been reported to possess bioactivities including anti-oxidative activity examined by luminol analogue L-012-dependent chemiluminescence method and anticoagulative activity determined using activated partial thromboplastin time reagent (Ueno et al., 2012; Li et al., 2017).

Selenium (Se) is an elementary trace element and is associated with the normal activities of organisms (Foster and Sumar, 1997). Se plays a critical role in various metabolic processes and is an important component of Se-dependent enzymes, such as glutathione peroxidase (GPx), which guards cells from serious oxidative damage induced by free radicals (Foster and Sumar, 1997). Many studies suggested that Se-containing compounds might be able to slow the progression of Alzheimer's disease (AD) due to their anti-oxidative effects and involvement in the molecular pathways of AD (Loef et al., 2011; Xie et al., 2018). The possible oxidation states of Se are selenate (+6), selenite (+4), selenium (0), and selenide (−2), and all these different oxidation states of Se can be assembled into a series of organic Se compounds such as dimethylselenide, trimethyselenium, selenomethionine, selenocysteine, and seleno-polysaccharides with sulphur being replaced by Se (Tinggi, 2003; Sun et al., 2014). Although selenosis in humans is very rare, endemic selenium toxicity in some parts of China and Australia still exists (Tinggi, 2003). Several studies have proved that low dose of Se is an effective anticarcinogen while high dose of Se can induce carcinogenesis, cytotoxicity, and even genotoxicity (Ramoutar and Brumaghim, 2007; Valdiglesias et al., 2010; Sun et al., 2014). As previously reported, organic Se compounds can improve the bio-availability of Se and possess fewer side effects than inorganic Se (Wang and Lovell, 1997). Seleno-polysaccharides, as a type of important organic Se compound, can be obtained by the reaction of Se with polysaccharide (Wei et al., 2015), or be extracted from plants (Zou et al., 2014) or fungi (Malinowska et al., 2009). Seleno-polysaccharides have exhibited bioactivities including antioxidation and neuroprotection that are superior to those of Se itself or Se-free polysaccharides (Yu et al., 2009; Wei et al., 2015).

β-amyloid (Aβ) is generated from amyloid precursor protein (APP) *via* cutting at β-site by APP-cleaving enzyme (β-secretase or BACE) and γ-secretase and consists of 36–43 amino acid residues (Lazarov and Demars, 2012). After cleavage, the Aβ peptide aggregates into oligomers and insoluble fibrils in brains. Aβ_1–42_ oligomers are suggested to be the most neurotoxic form (Pan et al., 2011). Aβ oligomers can induce the overproduction of reactive oxygen species (ROS) and cause dramatic oxidative damage to neurons, eventually leading to neuronal apoptosis and death (Kowall et al., 1991). The extracellular senile plaque formed by Aβ aggregation and precipitation is a primary histopathological characteristic of Alzheimer's disease (AD) which is a brain disease with serious neurodegeneration (Jana and Pahan, 2010). N2a-sw cell is the murine neuroblastoma N2a cell stably transfected with human Swedish mutant APP695, can thus overexpress APP and Aβ, and be an important cell model of AD.

We hypothesized and further confirmed that a new Se-containing compound, seleno-polymannuronate (Se-PM) obtained from the selenylation of alginate-derived PM by Na_2_SeO_3_ inherits the anti-oxidative bioactivity of Se-containing natural products and derivatives of alginate. For example, Se-PM decreased the ROS production through increasing the expressions of antioxidant enzymes including superoxide dismutase (SOD) and glutathione peroxidase (GPx) in N2a-sw cells (Zhu et al., 2013). Also, Se-PM inhibited ROS generation in lipopolysacharide (LPS)-stimulated RAW264.7 macrophages (Bi et al., 2018b). On the basis of the previous research work, we report the optimization of the preparation process of sulfated polymannuronate (S-PM), the compositional and structural characteristics of PM, S-PM, and Se-PM including the degree of sulfation, Se content and average molecular weight, and the inhibition of Aβ oligomer aggregation *in vitro* and neuroprotection effect in N2a-sw cells of Se-PM. Results from this study should be helpful to understand the nature of the new bioactivities caused by selenylation, and be useful to the development of new derivatives of alginate with better bioactivities.

## Methods

### Materials

Alginate, SO_3_-Py, and Na_2_SeO_3_ were purchased from Sigma-Aldrich (St. Louis, MO, USA). Foetal bovine serum (FBS), high-glucose DMEM, Opti-MEM, penicillin, streptomycin, and geneticin (G418) were provided by Gibco (Grand Island, NY, USA). Cell Counting Kit (CCK)-8, radioimmunoprecipitation assay (RIPA) buffer, and JC-1 fluorescence probe were provided by Beyotime Institute of Biotechnology (Jiangsu, China).

### Preparation of PM

PM was prepared from sodium alginate through hydrolysis using HCl and separation by pH 2.85 fractionation according to the method of Haug et al., 1967. In brief, 10 g alginate was dissolved in 500 mL 0.5 M HCl and then heated at 90°C for 7 h. After centrifuged at 3,500 rpm for 10 min, the white sediment was dissolved in 8% NaHCO_3_ and adjusted pH value to 2.85. After centrifugation at 3,500 rpm for 10 min, the solution was mixed with 95% ethyl alcohol (1:3, v:v) and precipitated overnight at 4°C. After centrifugation at 3,500 rpm for 10 min, the white sediment was dissolved in ultrapure water and PM was obtained after freeze drying by parameters of lyophilizer (Foring Technology Development (Beijing) Co., Ltd., Beijing, China). The homogeneity of PM was determined by circular dichroism (CD) (Jasco, Inc., Japan). As shown in **Supplemental Figure S1**, the peak and trough of CD spectrum of PM appeared at 200 and 216 nm respectively, indicating that the homogeneity of PM was over 90% according to literature (Morris et al., 1980).

### Preparation of S-PM

The sulfated derivative of PM was obtained by orthogonal experiment method under four different experimental conditions with various of temperatures and ratios of sulfating reagent (SO_3_-Py) to PM. PM (400 mg) and SO_3_-Py were each suspended in 20 mL of anhydrous dimethyl methanamide at room temperature (RT) with stirring in clean beakers, and then the SO_3_-Py solution was added dropwise into the PM solution. The mixture was stirred at 45°C or 60°C for 4 h and then cooled to RT. A precipitate was generated by the addition of absolute ethyl alcohol. After being washed three times with absolute ethyl alcohol, the precipitate was dissolved in ultrapure water, and the pH of the solution was adjusted to 7.0 using 1 M sodium hydroxide (NaOH) solution. Then, the solution was dialyzed (molecular weight cutoff ≥ 500 Da) against ultrapure water to remove residual sulfating reagent and potential degradation products until the conductivity remained constant. Sulfated PM (S-PM_1_ − S-PM_4_) with different DS values (**Table 1**) were obtained after freeze drying, and the samples were stored at −20°C until further analysis.

**Table 1:** Sulfur (S) content and DS of S-PM under four different experimental conditions.

Sample	SO_3_-Py : PM (w/w)	Temperature (°C)	Time (h)	S (%)	DS
S-PM_1_	4:1	45	4	7.31	0.48
S-PM_2_	4:1	60	4	8.41	0.58
S-PM_3_	6:1	45	4	9.48	0.69
S-PM_4_	6:1	60	4	11.86	0.97

### Preparation of Se-PM

The solution of S-PM was heated in the presence of Na_2_SeO_3_ (1:2, w/w) and excess barium chloride (BaCl_2_) in 5% HNO_3_ at 60°C for 8 h. After cooling, potassium sulfate (K_2_SO_4_) solution was gradually dropped into the reaction solution to remove excess Ba^2+^. Then, the reaction solution was centrifuged at 14,000 × g for 10 min to eliminate precipitated barium sulfate (BaSO_4_), and the pH of the clear reaction solution was adjusted to 7.0 using 1 M NaOH solution. Then, the solution was dialyzed (molecular weight cutoff ≥ 500 Da) against ultrapure water to remove additional chemical compounds until the conductivity remained constant and the solution did not turn red when reacted with ascorbic acid. Freeze drying afforded the powdered Se-PM, and the samples were stored at −20°C until further analyses.

### Measurement of the DS

The DS was used to quantify the amount of hydroxyl groups that had been replaced by sulfate groups, and it was estimated *via* the BaCl_2_-gelatin method (Dodgson and Price, 1962). Briefly, 10 mg of S-PM or Se-PM was dissolved in 1 mL of HCl (1 M) in an eppendorf tube and hydrolyzed at 100°C for 4 h. After centrifugation, 100 μL of the hydrolyzed solution was reacted with 900 μL of HCl (1 M) and 0.5 mL of BaCl_2_-gelatin (5 mg/mL) at RT for 20 min, and the absorbance values were determined at 500 nm. The ultrapure water was used as a blank and potassium sulphate (K_2_SO_4_) was used to construct calibration curves. The DS was calculated based on the following equation: DS = [(1.62 × S%)/(32 − 1.02 × S%)] and S% means the S percentage in the polysaccharide samples.

### Measurement of the Se Content

A 10-mg sample of Se-PM was digested with a mixture of perchloric acid (HClO_4_) and HNO_3_ (1:4, v/v) in a beaker overnight. Then, the reaction mixture was heated using an electrothermal furnace to generate a mass of white smoke. After cooling, the residue was dissolved in 6 M HCl and heated again. Then, the residue was dissolved in 10 mL of 0.5 M HCl. The Se content was measured by inductively coupled plasma-mass spectrometry (ICP-MS, Perkin-Elmer SCIEX, Norwalk, CT, USA) after digestion based on a calibration curves constructed using a Se standard solution [GBW(E)080215, 100 pg/mL] (National Standard Material Research Center, Beijing, China) (Zheng et al., 2016). The RF power was 1,100 W and the nebulizer used was Meinhard^®^. The plasma, auxiliary, and nebulizer gas flow rates were 20.0, 1.0, and 0.9 L/min, respectively.

### Measurement of the MW

The MW of the dissolved portions of PM, S-PM, and Se-PM were determined by an High performance liquid chromatography (HPLC) System (Agilent 1260, Palo Alto, CA, USA) coupled with a multi-angle laser scattering (MALLS) detector (Wyatt, Santa Barbara, CA, USA) and a refractive index (RI) detector (Agilent, Palo Alto, CA, USA). The value of dn/dc was set at 0.138 mL/g. The separation was performed on a chromatographic column (ACQUITY UPLC Protein BEH 125 Å, 1.7 µm, 4.6 × 300 mm, Waters, Milford, MA, USA) at 0.1 mL/min and 25°C. The injection volume was 20 μL, and the mobile phase was 20% methanol and 80% 80 mM ammonium acetate (NH_4_OAc).

### Fourier Transform Infrared (FT-IR) Spectroscopy Analysis

The chemical structures of PM, S-PM, and Se-PM were characterized using an FT-IR spectrophotometer (Thermo Scientific, Rochester, NY, USA) with a scanning range of 4,000 to 700 cm^−1^. Samples of the polysaccharide (2 mg) were mixed with potassium bromide (KBr) and pressed into thin discs for analysis.

### Thioflavin T (ThT) Fluorescence Analysis

To investigate the influence of polysaccharide samples on Aβ fibrillation, a ThT fluorescence-based analysis was performed. In brief, 20 µM freshly Aβ_1–42_ oligomer (Chinapeptides Co., Ltd., Guangdong, China) was diluted in PBS (pH 7.4) containing 20 mM ThT and 100 mM NaCl, and then 0.5 mg/mL Se-PM, PM, or 5 µM epigallocatechin gallate (EGCG) in PBS was added to the above system, respectively. One hundred fifty microliters of mixed solution was added to 96-well plates and incubated at 37°C for 10 h. The ThT fluorescence intensity of each group was recorded using a microplate reader (Fluoroskan Ascent FL, Thermo Scientific, Rochester, NY, USA) with 444/485 nm excitation/emission filters at different time points.

### Cell Culture

N2a cells were obtained from the Shanghai cell bank of the Chinese Academy of Sciences (Shanghai, China) and N2a-sw cells were the murine neuroblastoma N2a cell stably transfected with human Swedish mutant APP695 and as gifts provided by Prof. Xu Huaxi and Prof. Zhang Yunwu (Xiamen University, China). Both two cells cultured in 50% Opti-MEM and 44% DMEM supplemented with 5% FBS and 1% antibiotic (penicillin and streptomycin) in an incubator at 37°C with 5% CO_2_. There was additional 0.2% G418 in the N2a-sw cell culture medium.

In our previous study, the cytotoxicity of Se-PM was measured by CCK-8 assay. It showed that the viability of the N2a-sw cells increased significantly to 119% when the concentration of Se-PM reached 0.5 mg/mL (Zhu et al., 2013). Therefore, 0.5 mg/mL of polysaccharide samples were used in the following cell studies.

### Western Blot Analysis

The protein expression in N2a-sw cells after treated respectively by PM, S-PM, and Se-PM, was determined using Western blot analysis as described previously (Bi et al., 2019). The cells were lysed on ice with RIPA buffer containing a protease and phosphatase inhibitor cocktail (Selleck, Shanghai, China). The protein (20 μg) was resolved by 10% SDS-PAGE and transferred to a PVDF membrane. After blocking with 5% (w/v) skim milk, the membrane was probed with APP, BACE1, Bax, and Bcl-2 primary antibodies (Cell Signaling Technology, Beverly, MA, USA) or β-actin primary antibody (Proteintech, Hubei, China) at 4°C overnight and HRP-conjugated secondary antibody (Cell Signaling Technology, Beverly, MA, USA) at RT for 1 h. After rinsing, the proteins were visualized using an ECL kit (Thermo Scientific, Rochester, NY, USA), and the blot densities were quantified by Quantity One software.

### Immunofluorescence Analysis

After treatment, the cells were fixed with 4% formaldehyde and permeabilized with 0.2% (w/v) Triton X-100. Then, after blocking with 10% (w/v) goat serum, the cells were incubated with JC-1 fluorescence probe at 37°C for 30 min or cytochrome c primary antibody at 4°C overnight. After being washed, the cells incubated with JC-1 fluorescence probe and were directly visualized using laser scanning confocal microscopy (Carl Zeiss Jena Gmbh, Jena, Germany). The cells incubated with primary antibody were incubated with Alexa Fluor 596-conjugated secondary antibody and DAPI, which was used to label the nuclei at RT for another 2 h. After additional washes, the cells were observed by confocal microscopy. All the images were analyzed using the ImageJ software.

### Statistical Analysis

All the experiments were repeated at least three times. The data are presented as the means ± standard deviation and were analyzed using the two-tailed Student's t-test to determine any significant differences between the N2a cell group and N2a-sw cell group or between N2a-sw cell group and N2a-sw cell groups treated by different polysaccharides using GraphPad prism 5.01 software (GraphPad Software, Inc., La Jolla, CA, USA). A value of *p* < 0.05 was considered significant.

## Results and Discussion

### Chemical Characterization of PM, S-PM, and Se-PM

The sulfur (S) content and DS of S-PM_1_ to S-PM_4_ obtained from orthogonal experiments together with their experimental conditions are presented in **Table 1**. The results indicate that the S content in S-PM increased from 7.31% to 11.86% and that the DS of S-PM varied from 0.48 to 0.97. The hydroxyl groups on the PM were efficiently replaced with sulfate groups. The temperature and ratio of SO_3_-Py to PM were crucial factors that affected the S content and DS of S-PM, and the optimum conditions for further syntheses were determined to be SO_3_-Py and PM (6:1, w/w) reacting at 60°C for 4 h.

The molecular mass of polysaccharide samples was analyzed using HPLC-MALLS, and the results are listed in **Table 2**. The MWs of PM, S-PM, and Se-PM were 4.11 ± 0.27 kDa, 3.29 ± 0.06 kDa, and 2.36 ± 0.17 kDa respectively. With increasing chemical modification, the MWs of the polysaccharides obviously decreased potentially because degradation and depolymerization occurred simultaneously in the sulfation and selenylation processes.

**Table 2:** Chemical characterization analysis of polysaccharide samples.

Sample	MW (kDa)	DS	Se content (μg/g)
PM	4.11 ± 0.27	—	—
S-PM	3.29 ± 0.06	0.97	—
Se-PM	2.37 ± 0.17	0.56	437.25

In the selenylation process, Se might be in the form of -SeH or -SeO_3_ covalently bound to sites that were originally occupied by S. The Se content of Se-PM was 437.25 μg/g, and the DS of Se-PM decreased to 0.56 (**Table 2**). After selenylation, the DS in Se-PM decreased to 57.73% of that in S-PM, and the Se was detected to be 437.25 μg/g only in Se-PM, which is comparable with that reported for another kind of Se-polysaccharide prepared using nitric acid–sodium selenite (HNO_3_-Na_2_SeO_3_) method (Wei et al., 2015).

**Figure 1:**
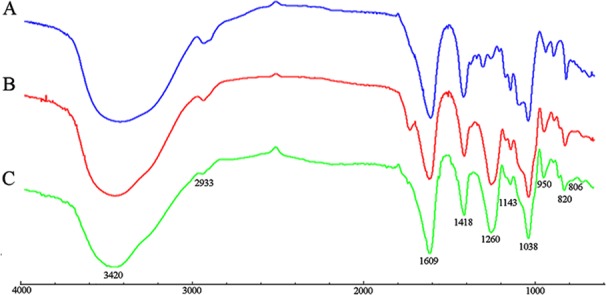
FT-IR spectra of **(A)** PM, **(B)** S-PM, and **(C)** Se-PM.

The FT-IR spectra of PM, S-PM, and Se-PM in the range of 4,000 to 700 cm^−1^ are presented in **Figure 1**. The typical FT-IR signals of polysaccharides were observed, and the general profiles of each sample are similar. The characteristic at 3,420 and 2,933 cm^−1^ were assigned to the asymmetric stretching vibration of -OH and -CH groups, respectively. The absorption bands at 1,609 and 1,418 cm^−1^ were due to the symmetric and asymmetric stretching vibrations of -COOH groups (Gomez Bujedo et al., 2004). Three absorption s from 1,200 to 1,000 cm^−1^ were indicative of pyranoid saccharides, and the weak absorption at 820 cm^−1^ was unique to the mannuronic acid residues (Leal et al., 2008). However, there are some differences between the FT-IR spectra of PM, S-PM, and Se-PM. The existence of sulfate groups in S-PM and Se-PM was confirmed. As presented in **Figure 1B**, the specific absorption at 1,260 and 806 cm^−1^ were related to the asymmetric stretching vibration of sulfate esters (–S = O) and to the -C-O-S symmetric stretching vibration, respectively (Vasconcelos et al., 2013), which suggested that the sulfated derivative was successfully prepared. As shown in **Figure 1C**, the spectrum of Se-PM was similar to that of S-PM. The typical FT-IR signals of NaSeO_3_ appear at 790 and 730 cm^−1^ (Torrie, 2011). No band in this region was seen in **Figure 1C**, which indicated that as the reactant of selenylation of S-PM, the free Na_2_SeO_3_ was removed in the previous dialysis purification process. Considering that the measured Se content in the synthesized Se-PM was 437.25 μg/g while the measured S content in Se-PM decreased, implying that some bound S was replaced by Se, we thus speculate that the selenylated derivative was successfully synthesized and that Se might be covalently bound to the polysaccharide.

### Se-PM Inhibits Aβ_1–42_ Aggregation *In Vitro*

**Figure 2:**
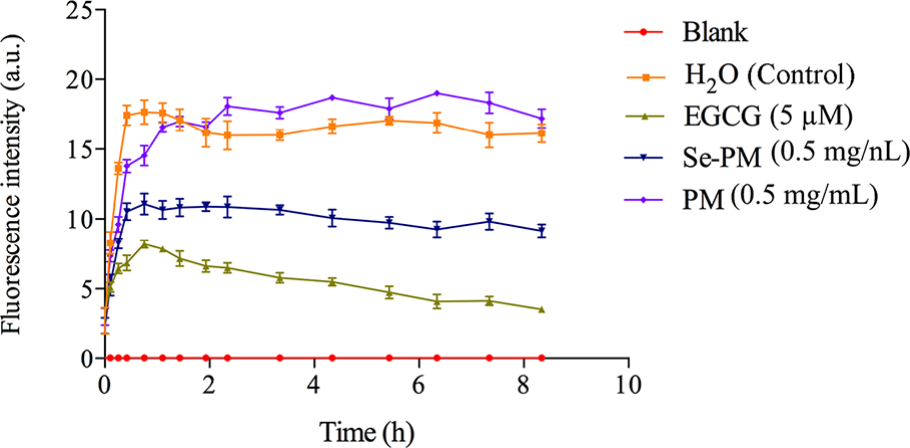
Effects of Se-PM on Aβ_1–42_ aggregation *in vitro*.

The inhibition of Se-PM or PM on Aβ_1–42_ aggregation was examined by ThT fluorescence assay, which is a valuable method for a real-time evaluation of formation of Aβ aggregation *in vitro* (Hu et al., 2004). As shown in **Figure 2**, the progress in fibrillization of Aβ_1–42_ was continuously monitored by the increase in fluorescence of the dye ThT in the incubation mixture. A fast increase in ThT fluorescence was observed when mixed with Aβ_1–42_. After incubated with 0.5 mg/mL Se-PM, the maximal level of ThT fluorescence was obviously less by about 35% than that in control group, indicating that Se-PM suppressed the aggregation of Aβ_1–42_, while incubation with 0.5 mg/mL PM showed no influence on Aβ_1–42_ aggregation. The EGCG was chosen as a positive control.

After sheared from APP, the Aβ peptide would aggregate into oligomers and insoluble fibrils (Lazarov and Demars, 2012). It has been reported that Aβ oligomers were more toxic than Aβ monomers or fibrils, and Aβ_1–42_ oligomers are suggested to be the most neurotoxic form (Pan et al., 2011; Benilova et al., 2012). The inhibition of Aβ_1–42_ aggregation by Se-PM might be one of the most important reasons for Se-PM to promote N2a-sw cell survival, exhibited as an increase in its cell viability.

### Se-PM Suppresses Aβ Pathway in N2a-sw Cells

**Figure 3:**
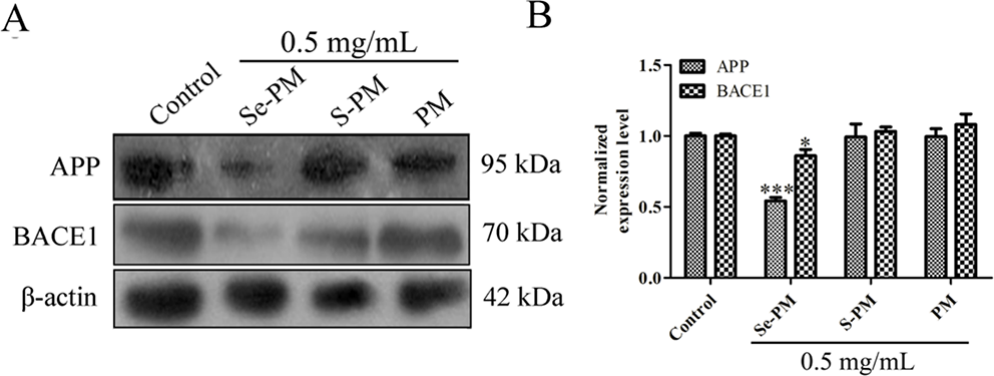
Effects of polysaccharide samples on the APP and BACE1 expression levels in N2a-sw cells: **(A)** immunoblot, **(B)** statistical analysis. * *p* < 0.05, *** *p* < 0.001.

Aberrant accumulation of Aβ leads to the formation of Aβ plaques in brains, triggering the serious toxicity on nervous system (Xie et al., 2017). The total Aβ level in neurocytes is maintained in a dynamic equilibrium between generation and clearance, and the expression of Aβ sheared from APP in N2a-sw cells is much higher than that in N2a cells (Jana and Pahan, 2010; Zhang et al., 2017). Next, the Aβ pathway affected by Se-PM in N2a-sw cells was confirmed. First, the expression levels of APP in N2a-sw cells after treatment with Se-PM, S-PM, or PM were measured using Western blot analysis. Se-PM treatment effectively decreased APP expression compared with the control group. However, the S-PM-treated group and the PM-treated group did not have significant effect on APP expression (**Figures 3A, B**).

BACE1 causes the initiation of Aβ formation in the amyloid genic pathway, which shears APP to produce the N-terminus of Aβ (β-CTF), and then the β-CTF is cleaved by γ-secretase to generate the mature Aβ peptide (Xie et al., 2017). The expression levels of BACE1 in the N2a-sw cells were confirmed using Western blot analysis after treatment with Se-PM, S-PM, or PM. The results presented in **Figures 3A, B** show that BACE1 levels in N2a-sw cells were markedly reduced in the Se-PM-treated group while BACE1 levels in N2a-sw cells didn't change statistically after treated by S-PM and PM, respectively.

APP was overexpressed in N2a-sw cells due to being transferred the human APP695-Swedish gene. Therefore, the mechanism by which Se-PM can decrease protein expression of APP is warrant further study, including determining the APP mRNA expression, autophagy, and the ubiquitin-proteasome degradation pathway. In addition, Se-PM having significantly reduced the expression of APP and BACE1 while S-PM and PM could not suggest that the Se in the polysaccharide might be a vital factor. A number of studies have reported that Se-containing compounds possess more potent activities than Se-free compounds for neuroprotection (Steinbrenner et al., 2006; Loef et al., 2011). In our previous study, Se-PM was shown to markedly suppress the nuclear translocation of nuclear factor-κB (NF-κB) (Bi et al., 2018b). Specifically, the promoter region of BACE1 has numerous NF-κB binding sites (Sambamurti et al., 2004); thus, Se-PM might reduce the transcription of the BACE1 gene *via* inhibition of NF-κB binding to DNA, decreasing the expression of BACE1.

### Se-PM Inhibits Cell Apoptosis in N2a-sw Cells

**Figure 4:**
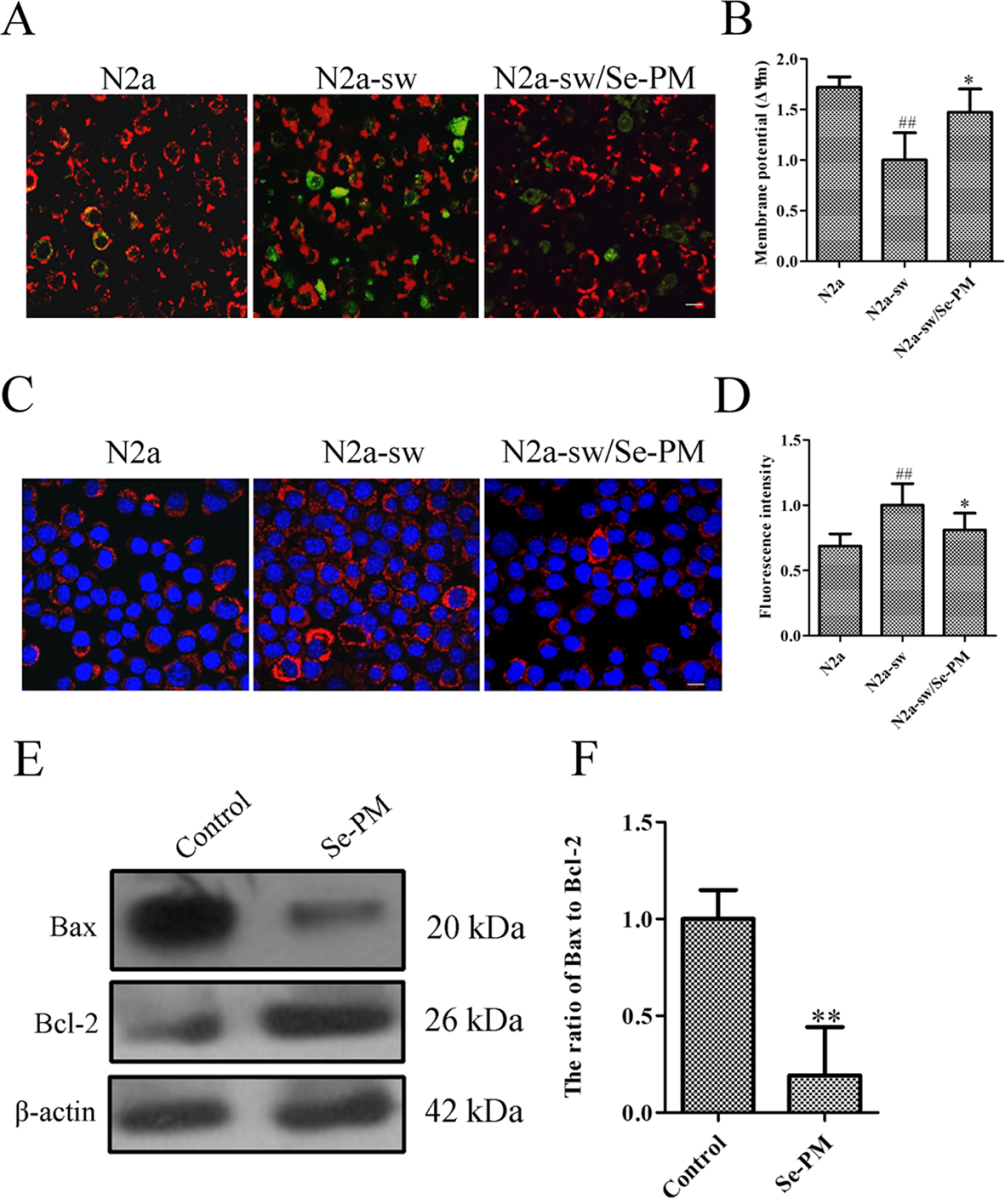
Effects of Se-PM (0.5 mg/mL) on cell apoptosis in N2a-sw cells. **(A, B)** Immunofluorescence analysis of the mitochondrial membrane potential **(A)** and statistical analysis **(B)**. **(C, D)** Immunofluorescence analysis of cytochrome c release **(C)** and statistical analysis **(D)**. **(E, F)** Protein expression levels of Bax/Bcl-2, detected by Western blot analysis **(E)** and statistical analysis **(F)**. Indicates significant differences between the N2a-sw cells and N2a cells, ^##^*p* < 0.01. *Indicates significant differences between the Se-PM-treated groups and control in N2a-sw cells, * *p* < 0.05. ** *p* < 0.01. Scale bar = 20 μm.

Oxidative stress induced by the aberrant production of APP and Aβ oligomers is a strong trigger for neurocyte apoptosis in nervous system (Huang et al., 2004). Reduction of the mitochondrial membrane potential indicates an increase in permeability and the release of some substances, such as cytochrome c and apoptosis-inducing factors, to the cytoplasm (Petit et al., 1998). The effect of Se-PM on the mitochondrial membrane potential was determined using JC-1, which is a highly sensitive metachromatic and lipophilic cationic dye-based probe. JC-1 accumulates in the mitochondrial matrix as a monomer and produces green fluorescence at low membrane potentials, and it forms “J-aggregates” and produces red fluorescence at high membrane potentials (Trimmer et al., 2000). An obvious increase in green fluorescence was observed in N2a-sw cells compared with N2a cells, which indicated that the mitochondrial membrane potential in N2a-sw cells was much lower than that in N2a cells. The green fluorescence in N2a-sw cells was significantly weakened after treatment with Se-PM, suggesting that Se-PM treatment could effectively increase the mitochondrial membrane potential in N2a-sw cells (**Figure 4A**). Furthermore, the mitochondrial membrane potential of each group was statistically analyzed by ImageJ software. The mitochondrial membrane potential in N2a-sw cells was 58.17% of that in N2a cells, and it increased 1.47-fold with Se-PM treatment compared with untreated N2a-sw cells (**Figure 4B**).

Next, the expression levels of cytochrome c in N2a cells and N2a-sw cells were determined using immunofluorescence analysis. The levels of cytochrome c (red) in N2a-sw cells were much higher than those in N2a cells, whereas the level was visibly reduced in N2a-sw cells after treatment with Se-PM (**Figure 4C**). The fluorescence intensities of cytochrome c were measured by ImageJ software. The expression of cytochrome c in N2a-sw cells was shown to be 1.46-fold of that in N2a cells, and it was reduced to 81% by Se-PM treatment compared with that in untreated N2a-sw cells (**Figure 4D**).

The Bcl-2 family has been proved to regulate apoptosis through the mitochondrial pathway (Robertson and Orrenius, 2000). Bax and Bcl-2 are representative pro-apoptotic and anti-apoptotic members of this family, respectively (Robertson and Orrenius, 2000). The ratio of Bax to Bcl-2 is a decisive factor in the induction of apoptosis, and the balance between the expression levels of Bax and Bcl-2 is a critical key for cell death or survival (Jia et al., 2015). N2a-sw cells were treated with Se-PM for 24 h, and then the Bax and Bcl-2 expression levels were determined by Western blot analysis. The expression of Bax in N2a-sw cells was obviously reduced, and the Bcl-2 expression level was considerably increased (**Figure 4E**). The band density analysis showed that there was a significant decrease in the ratio of Bax to Bcl-2 in N2a-sw cells after treatment with Se-PM (**Figure 4F**), indicating that Se-PM could inhibit apoptosis of N2a-sw cells induced by overexpressed APP and Aβ. Se, as a primary trace element, plays an important role in anti-oxidation and inhibition of the ROS over-expression in organisms. It is well known that ROS is a key factor to mediate cell apoptosis (Zhou et al., 2009). It was also reported that seleno-polysaccharides obtained from *Radix hedysari* possessed significant anti-apoptosis activity in Aβ_21–45_-induced SH-SY5Y cells (Wei et al., 2015).

Se normally plays a critical role in neuroprotection. It could guard cells from serious oxidative damage, and seleno-polysaccharide, as an important organic Se material, has exhibited excellent bioactivities. Seleno-polysaccharide synthesized from *R. hedysari* polysaccharide and Na_2_SeO_3_ presents notable neuroprotective effects against oxidative damage in Aβ_21–45_-triggered SH-SY5Y cells (Wei et al., 2015). Seleno-polysaccharide isolated from *Coprinus comatus* show dramatic hypoglycaemic and antioxidant activities in diabetic mice (Yu et al., 2009). Our previous studies have proved that Se-PM could effectively reduce ROS production in N2a-sw cells (Zhu et al., 2013) and LPS-triggered RAW264.7 cells (Bi et al., 2018a). In this study, we found that Se-PM presented excellent neuroprotection bioactivities and perfectly combined the properties of PM and Se. However, Se-PM may not be able to function through the blood-brain barrier (BBB). To verify whether Se-PM could transport across the BBB, we inoculated BALB/c mice with 100 μL of Se-PM (20 mg/mL) *via* a tail vein injection. The Se concentration in the brain increased by approximately 19% at 2 h after the Se-PM injection, and then tended to balance (**Supplemental Figure S2**), suggesting that Se-PM could cross the BBB to serve its function.

ROS have been reported to serve as important chemical messengers and play a key role in cell homeostasis, proliferation, and growth (Jia et al., 2015). However, ROS become a powerful trigger of cell apoptosis when the production of ROS is out of control (Su et al., 2005). Recent studies have suggested that impaired mitochondrial function is induced by Aβ accumulation in mitochondria, and the binding of Aβ to mitochondrial membranes is also an important inducer of AD, and it can induce neuronal dysfunction and apoptosis with ROS overproduction (Manczak et al., 2006). In the present study, Se-PM treatment could significantly decrease Aβ aggregation *in vitro* (**Figure 2**) and reduce APP and BACE1 expression in N2a-sw cells (**Figure 3**). Combined with the experimental results from our previous study that Se-PM significantly decrease ROS production in N2a-sw cells (Zhu et al., 2013), we speculated that the levels of Aβ oligomers in N2a-sw cells could also be reduced by Se-PM treatment.

An increase in ROS has been implicated in the induction of apoptosis because it decreases the mitochondrial membrane potential and promotes the release of cytochrome c to facilitate caspase activation (Kagan et al., 2005). Cytochrome c release is regulated by Bcl-2, which is an antagonist of the proapoptotic activity of Bax and promotes cell survival *via* attenuating Bax translocation from the cytosol to mitochondria (Hou et al., 2003). Se-PM treatment could significantly alleviate the expression of cytochrome c and reduce the ratio of Bax to Bcl-2 in N2a-sw cells (**Figures 4C–F**). These results suggest that Se-PM treatment can markedly inhibit the cell apoptosis induced by the overexpression of APP or Aβ oligomers and dramatically promote cell survival of N2a-sw cells.

## Conclusion

In summary, we successfully synthesized a Se-containing compound, Se-PM using alginate-derived PM and Na_2_SeO_3_, and its neuroprotection as well as mechanism were evaluated in this study.

From our study, for the FT-IR spectrum of Se-PM, the typical IR signals of NaSeO_3_ (790 and 730 cm^−1^) did not appear, which indicated that the free Na_2_SeO_3_ was removed in the previous dialysis purification process. In addition, considering that the measured Se content in the synthesized Se-PM was 437.25 μg/g while the measured S content in Se-PM decreased, it is sure that some Se was bound to polysaccharides although the type of chemical bond of Se with polysaccharides is unclear. Furthermore, the bioactivities of PM, S-PM, and Se-PM are significant different, further confirming our speculation that Se may have replaced S and bound into polysaccharides.

Se-PM remarkably inhibited Aβ_1–42_ oligomer aggregation *in vitro* and suppressed APP and BACE1 expression in N2a-sw cells. Moreover, Se-PM attenuated the expression of cytochrome c and enhanced the mitochondrial membrane potential in N2a-sw cells. Se-PM also remarkably decreased the ratio of Bax to Bcl-2. All these suggested that Se-PM could reduce cell apoptosis and promote cell survival of N2a-sw cells. These findings should be helpful to understand the mechanism for the new activities caused by Se supplementation in drugs, and may provide a pharmacological basis for the application of Se-derivatives of polysaccharide as original marine drugs in neuroprotection, to improve human health.

## Data Availability Statement

The datasets generated for this study are available on request to the corresponding authors.

## Author Contributions

XX, QL, ZZ, DB and XfL conceived and designed the project. DB, XfL, XtL, ZL, TL, and HL performed majority of the experiments. DB, LY and TL performed data analyses. XX, QL, DB, XfL, HX, and ZH wrote the manuscript. XX, DB and XfL revised the paper. XX, QL and ZZ supervised the paper.

## Funding

This work was supported financially by the National Key R&D Program of China (2018YFD0901106), the National Natural Science Foundation of China (31871734 and 21877081), the Guangdong Natural Science Foundation (2018A0303130054, 2016A030313052 and 2018A030313507), the Science and Technology Innovation Commission of Shenzhen (JCYJ20170818143107733, JCYJ20180507182405562, JCYJ20180305124211995 and JCYJ20180305125619343), the Guangdong Natural Science Foundation for Major Cultivation Project (2018B030336001) and the State Oceanic Administration 13th Five-Year Marine Special Fund for Demonstration City, Shenzhen Basic Research Project (Subject Layout Project) to ZH.

The authors thank the Beijing Advanced Innovation Center for Food Nutrition and Human Health of Beijing Technology and Business University (BTBU), and also thank the Instrumental Analysis Center of Shenzhen University (Xili Campus) for their assistance in our experiments.

## Conflict of Interest

The authors declare that the research was conducted in the absence of any commercial or financial relationships that could be construed as a potential conflict of interest.

## References

[B1] Benilova I. Karran E. De Strooper B. (2012). The toxic Aβ oligomer and Alzheimer's disease: an emperor in need of clothes. Nat. Neurosci. 15, 349. 10.1038/nn.3028 22286176

[B2] Bi D. Zhou R. Cai N. Lai Q. Han Q. Peng Y. (2017). Alginate enhances Toll-like receptor 4-mediated phagocytosis by murine RAW264. 7 macrophages. Int. J. Biol. Macromol. 105, 1446–1454. 10.1016/j.ijbiomac.2017.07.129 28739412

[B3] Bi D. Lai Q. Cai N. Li T. Zhang Y. Han Q. (2018a). Elucidation of the molecular-mechanisms and *in vivo* evaluation of the anti-inflammatory effect of alginate-derived Seleno-polymannuronate. J. Agric. Food Chem. 66, 2083–2091. 10.1021/acs.jafc.7b05719 29406745

[B4] Bi D. Lai Q. Han Q. Cai N. He H. Fang W. (2018b). Seleno-polymannuronate attenuates neuroinflammation by suppressing microglial and astrocytic activation. J. Funct. Foods 51, 113–120. 10.1016/j.jff.2018.10.010

[B5] Bi D. Lai Q. Li X. Cai N. Li T. Fang W. (2019). Neuroimmunoregulatory potential of seleno-polymannuronate derived from alginate in lipopolysaccharide-stimulated BV2 microglia. Food Hydrocolloids 87, 925–932. 10.1016/j.foodhyd.2018.09.013

[B6] Dodgson K. Price R. (1962). A note on the determination of the ester sulphate content of sulphated polysaccharides. Biochem. J. 84, 106–110. 10.1042/bj0840106 13886865 PMC1243628

[B7] Fang W. Bi D. Zheng R. Cai N. Xu H. Zhou R. (2017). Identification and activation of TLR4-mediated signalling pathways by alginate-derived guluronate oligosaccharide in RAW264. 7 macrophages. Sci. Rep. 7, 1663. 10.1038/s41598-017-01868-0 28490734 PMC5431981

[B8] Foster L. Sumar S. (1997). Selenium in health and disease: a review. Crit. Rev. In Food Sci. Nutr. 37, 211–228. 10.1080/10408399709527773 9143818

[B9] Gomez Bujedo S. Fleury E. Vignon M. R. (2004). Preparation of cellouronic acids and partially acetylated cellouronic acids by TEMPO/NaClO oxidation of water-soluble cellulose acetate. Biomacromolecules 5, 565–571. 10.1021/bm034405y 15003022

[B10] Haug A. Larsen B. Smidsrod O. (1967). Studies on the sequence of uronic acid residues in alginic acid. Acta Chem. Scand. 21, 691–704. 10.3891/acta.chem.scand.21-0691

[B11] Hou Q. Cymbalyuk E. Hsu S. C. Xu M. Hsu Y. T. (2003). Apoptosis modulatory activities of transiently expressed Bcl-2: roles in cytochrome c release and Bax regulation. Apoptosis 8, 617–629. 10.1023/A:1026187526113 14739607

[B12] Hu J. Geng M. Li J. Xin X. Wang J. Tang M. (2004). Acidic oligosaccharide sugar chain, a marine-derived acidic oligosaccharide, inhibits the cytotoxicity and aggregation of amyloid beta protein. J. Pharmacol. Sci. 95, 248–255. 10.1254/jphs.FPJ04004X 15215650

[B13] Huang X. Moir R. D. Tanzi R. E. Bush A. I. Rogers J. T. (2004). Redox-active metals, oxidative stress, and Alzheimer's disease pathology. Ann. New Y. Acad. Sci. 1012, 153–163. 10.1196/annals.1306.012 15105262

[B14] Jana A. Pahan K. (2010). Fibrillar amyloid-β-activated human astroglia kill primary human neurons *via* neutral sphingomyelinase: implications for Alzheimer's disease. J. Neurosci. 30, 12676–12689. 10.1523/JNEUROSCI.1243-10.2010 20861373 PMC3020912

[B15] Jia G. Wang Q. Wang R. Deng D. Xue L. Shao N. (2015). Tubeimoside-1 induces glioma apoptosis through regulation of Bax/Bcl-2 and the ROS/Cytochrome C/Caspase-3 pathway. Oncotargets Ther. 8, 303–311. 10.2147/OTT.S76063 PMC4321652 25674005

[B16] Kagan V. E. Tyurin V. A. Jiang J. Tyurina Y. Y. Ritov V. B. Amoscato A. A. (2005). Cytochrome c acts as a cardiolipin oxygenase required for release of proapoptotic factors. Nat. Chem. Biol. 1, 223–232. 10.1038/nchembio727 16408039

[B17] Kowall N. W. Beal M. F. Busciglio J. Duffy L. K. Yankner B. A. (1991). An *in vivo* model for the neurodegenerative effects of beta amyloid and protection by substance P. Proc. Natl. Acad. Sci. 88, 7247–7251. 10.1073/pnas.88.16.7247 1714596 PMC52271

[B18] Lazarov O. Demars M. P. (2012). All in the family: how the APPs regulate neurogenesis. Front. In Neurosci. 6, 81. 10.3389/fnins.2012.00081 PMC3366480 22675290

[B19] Leal D. Matsuhiro B. Rossi M. Caruso F. (2008). FT-IR spectra of alginic acid block fractions in three species of brown seaweeds. Carbohydr. Res. 343, 308–316. 10.1016/j.carres.2007.10.016 18048014

[B20] Lee K. Y. Mooney D. J. (2012). Alginate: properties and biomedical applications. Prog. In Polymer Sci. 37, 106. 10.1016/j.progpolymsci.2011.06.003 PMC3223967 22125349

[B21] Li Q. Zeng Y. Wang L. Guan H. Li C. Zhang L. (2017). The heparin-like activities of negatively charged derivatives of low-molecular-weight polymannuronate and polyguluronate. Carbohydr. Polymer 155, 313–320. 10.1016/j.carbpol.2016.08.084 27702517

[B22] Loef M. Schrauzer G. N. Walach H. (2011). Selenium and Alzheimer's disease: a systematic review. J. Alzheimer's Dis. 26, 81–104. 10.3233/JAD-2011-110414 21593562

[B23] Malinowska E. Krzyczkowski W. Herold F. Łapienis G. Ślusarczyk J. Suchocki P. (2009). Biosynthesis of selenium-containing polysaccharides with antioxidant activity in liquid culture of Hericium erinaceum. Enzyme Microbial Technol. 44, 334–343. 10.1016/j.enzmictec.2008.12.003

[B24] Manczak M. Anekonda T. S. Henson E. Park B. S. Quinn J. Reddy P. H. (2006). Mitochondria are a direct site of A beta accumulation in Alzheimer's disease neurons: implications for free radical generation and oxidative damage in disease progression. Hum. Mol. Genet. 15, 1437–1449. 10.1093/hmg/ddl066 16551656

[B25] Morris E. R. Rees D. A. Thom D. (1980). Characterisation of alginate composition and block-structure by circular dichroism. Carbohydr. Res. 81, 305–314. 10.1016/S0008-6215(00)85661-X

[B26] Pan X. Zhu Y. Lin N. Zhang J. Ye Q. Huang H. (2011). Microglial phagocytosis induced by fibrillar β-amyloid is attenuated by oligomeric β-amyloid: implications for Alzheimer's disease. Mol. Neurodegeneration 6, 45. 10.1186/1750-1326-6-45 PMC3149591 21718498

[B27] Petit P. Goubern M. Diolez P. Susin S. Zamzami N. Kroemer G. (1998). Disruption of the outer mitochondrial membrane as a result of large amplitude swelling: the impact of irreversible permeability transition. FEBS Lett. 426, 111–116. 10.1016/S0014-5793(98)00318-4 9598989

[B28] Ramoutar R. R. Brumaghim J. L. (2007). Effects of inorganic selenium compounds on oxidative DNA damage. J. Inorg. Biochem. 101, 1028–1035. 10.1016/j.jinorgbio.2007.03.016 17531322

[B29] Robertson J. D. Orrenius S. (2000). Molecular mechanisms of apoptosis induced by cytotoxic chemicals. Crit. Rev. In Toxicol. 30, 609–627. 10.1080/10408440008951122 11055838

[B30] Sambamurti K. Kinsey R. Maloney B. Ge Y.-W. Lahiri D. K. (2004). Gene structure and organization of the human β-secretase (BACE) promoter. FASEB J. 18, 1034–1036. 10.1096/fj.03-1378fje 15059975

[B31] Steinbrenner H. Alili L. Bilgic E. Sies H. Brenneisen P. (2006). Involvement of selenoprotein P in protection of human astrocytes from oxidative damage. Free Radical Biol. Med. 40, 1513–1523. 10.1016/j.freeradbiomed.2005.12.022 16632112

[B32] Su Y. T. Chang H. L. Shyue S. K. Hsu S. L. (2005). Emodin induces apoptosis in human lung adenocarcinoma cells through a reactive oxygen species-dependent mitochondrial signaling pathway. Biochem. Pharmacol. 70, 229–241. 10.1016/j.bcp.2005.04.026 15941563

[B33] Sun H.-J. Rathinasabapathi B. Wu B. Luo J. Pu L.-P. Ma L. Q. (2014). Arsenic and selenium toxicity and their interactive effects in humans. Environ. Int. 69, 148–158. 10.1016/j.envint.2014.04.019 24853282

[B34] Tinggi U. (2003). Essentiality and toxicity of selenium and its status in Australia: a review. Toxicol. Lett. 137, 103–110. 10.1016/S0378-4274(02)00384-3 12505436

[B35] Torrie B. H. (2011). Raman and infrared spectra of Na2SeO3, NaHSeO3, H2SeO3, and NaH3(SeO3). Can. J. Phys. 51, 610–615. 10.1139/p73-080

[B36] Trimmer P. Swerdlow R. Jk Keeney P. Bennett J. J. Miller S. Davis R. (2000). Abnormal mitochondrial morphology in sporadic Parkinson's and Alzheimer's disease cybrid cell lines. Exp. Neurol. 162, 37–50. 10.1006/exnr.2000.7333 10716887

[B37] Tusi S. K. Khalaj L. Ashabi G. Kiaei M. Khodagholi F. (2011). Alginate oligosaccharide protects against endoplasmic reticulum-and mitochondrial-mediated apoptotic cell death and oxidative stress. Biomaterials 32, 5438–5458. 10.1016/j.biomaterials.2011.04.024 21543116

[B38] Ueno M. Hiroki T. Takeshita S. Jiang Z. Kim D. Yamaguchi K. (2012). Comparative study on antioxidative and macrophage-stimulating activities of polyguluronic acid (PG) and polymannuronic acid (PM) prepared from alginate. Carbohydr. Res. 352, 88–93. 10.1016/j.carres.2012.02.005 22402099

[B39] Valdiglesias V. Pásaro E. Méndez J. Laffon B. (2010). *In vitro* evaluation of selenium genotoxic, cytotoxic, and protective effects: a review. Arch. Toxicol. 84, 337–351. 10.1007/s00204-009-0505-0 20033805

[B40] Vasconcelos A. F. D. Dekker R. F. H. Barbosa A. M. Carbonero E. R. Silveira J. L. M. Glauser B. (2013). Sulfonation and anticoagulant activity of fungal exocellular β-(1→6)- d -glucan (lasiodiplodan). Carbohydr. Polymer 92, 1908–1914. 10.1016/j.carbpol.2012.10.034 23399236

[B41] Wang C. Lovell R. T. (1997). Organic selenium sources, selenomethionine and selenoyeast, have higher bioavailability than an inorganic selenium source, sodium selenite, in diets for channel catfish (Ictalurus punctatus). Aquaculture 152, 223–234. 10.1016/S0044-8486(96)01523-2

[B42] Wei D. Chen T. Yan M. Zhao W. Li F. Cheng W. (2015). Synthesis, characterization, antioxidant activity and neuroprotective effects of selenium polysaccharide from Radix hedysari. Carbohydr. Polymer 125, 161–168. 10.1016/j.carbpol.2015.02.029 25857971

[B43] Xie Y. Tan Y. Zheng Y. Du X. Liu Q. (2017). Ebselen ameliorates β-amyloid pathology, tau pathology, and cognitive impairment in triple-transgenic Alzheimer's disease mice. J. Biol. Inorg. Chem. 22, 851–865. 10.1007/s00775-017-1463-2 28502066

[B44] Xie Y. Liu Q. Zheng L. Wang B. Qu X. Ni J. (2018). Se-Methylselenocysteine ameliorates neuropathology and cognitive deficits by attenuating oxidative stress and metal dyshomeostasis in alzheimer model mice. Mol. Nutr. Food Res., 1800107. 10.1002/mnfr.201800107 29688618

[B45] Xu X. Bi D. Wu X. Wang Q. Wei G. Chi L. (2014a). Unsaturated guluronate oligosaccharide enhances the antibacterial activities of macrophages. FASEB J. 28, 2645–2654. 10.1096/fj.13-247791 24599964

[B46] Xu X. Wu X. Wang Q. Cai N. Zhang H. Jiang Z. (2014b). Immunomodulatory effects of alginate oligosaccharides on murine macrophage RAW264. 7 cells and their structure–activity relationships. J. Agric. Food Chem. 62, 3168–3176. 10.1021/jf405633n 24628671

[B47] Yu J. Cui P. Zeng W. Xie X. Liang W. Lin G. (2009). Protective effect of selenium-polysaccharides from the mycelia of Coprinus comatus on alloxan-induced oxidative stress in mice. Food Chem. 117, 42–47. 10.1016/j.foodchem.2009.03.073

[B48] Zhang Z. Wu Q. Chen C. Zheng R. Chen Y. Liu Q. (2017). Selenomethionine attenuates the Amyloid-β level by both inhibiting Amyloid-β production and modulating autophagy in neuron-2a/AβPPswe cells. J. Alzheimer's Dis. 59, 591–602. 10.3233/JAD-170216 28671121

[B49] Zheng L. Zhu H.-Z. Wang B.-T. Zhao Q.-H. Du X.-B. Zheng Y. (2016). Sodium selenate regulates the brain ionome in a transgenic mouse model of Alzheimer's disease. Sci. Rep. 6, 39290. 10.1038/srep39290 28008954 PMC5180247

[B50] Zhou Y. Zhang S. Liu C. Cai Y. (2009). The protection of selenium on ROS mediated-apoptosis by mitochondria dysfunction in cadmium-induced LLC-PK1 cells. Toxicol. In Vitro 23, 288–294. 10.1016/j.tiv.2008.12.009 19135140

[B51] Zhou R. Shi X. Bi D. Fang W. Wei G. Xu X. (2015a). Alginate-derived oligosaccharide inhibits neuroinflammation and promotes microglial phagocytosis of β-amyloid. Marine Drugs 13, 5828–5846. 10.3390/md13095828 26389923 PMC4584357

[B52] Zhou R. Shi X. Gao Y. Cai N. Jiang Z. Xu X. (2015b). Anti-inflammatory activity of guluronate oligosaccharides obtained by oxidative degradation from alginate in lipopolysaccharide-activated murine macrophage RAW 264.7 cells. J. Agric. Food Chem. 63, 160–168. 10.1021/jf503548a 25483391

[B53] Zhu Z. Liu Q. Chen P. Xu X. Ni J. Yang S. (2013). Seleno-polymannuronate synthesis and resistance to oxidation and apoptosis in Alzheimer's disease cells. Chem. J. Chin. 34, 115–122.

[B54] Zou Y. Zhao T. Mao G. Zhang M. Zheng D. Feng W. (2014). Isolation, purification and characterisation of selenium-containing polysaccharides and proteins in selenium-enriched Radix puerariae. J. Sci. Food Agric. 94, 349–358. 10.1002/jsfa.6366 24037994

