# Supplementary material for: Characterization and Neuroprotection Potential of Seleno-Polymannuronate
Source: Front Pharmacol. 2020 Feb 20;11:21. doi: 10.3389/fphar.2020.00021 (PMC7044149; doi:10.3389/fphar.2020.00021)
Supplement: Supplementary file 1 [file DataSheet_1.pdf]

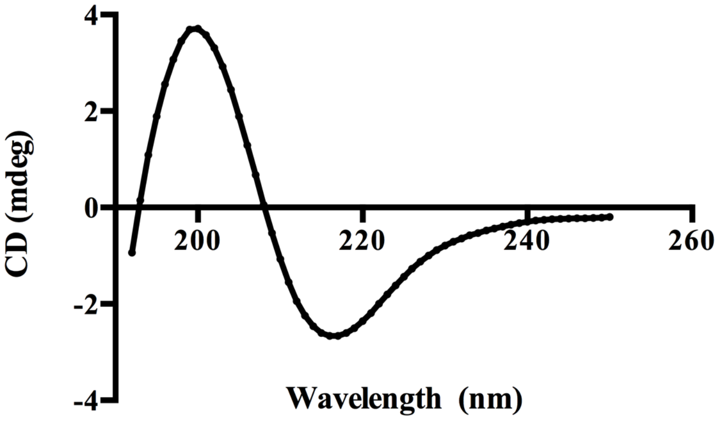

Figure S1. CD spectrum of PM.

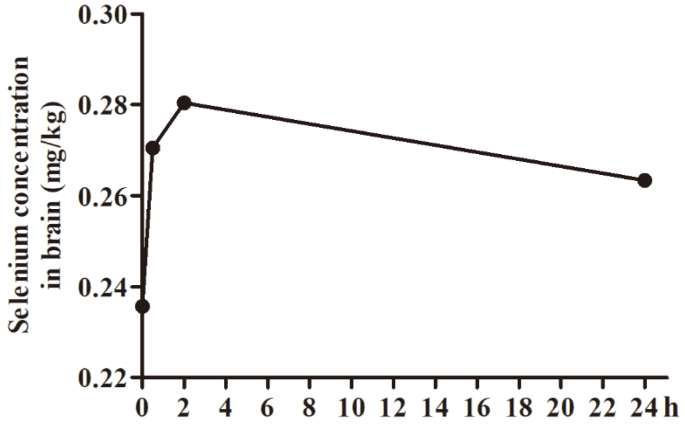

Figure S2. The change of Se content in the mice brain after caudal vein injection with Se-PM.
